# Application of a staged health education pathway checklist in perioperative care of patients with esophageal cancer

**DOI:** 10.1186/s12876-025-04351-7

**Published:** 2025-10-22

**Authors:** Qinyun Li, Chenxi Zeng, Mengxia Qin, Wenbin Zou, Xiaoying Hou

**Affiliations:** Department of Thoracic Surgery, Tongji Hospital, Tongji Medical College, Huazhong University of Science and Technology, 1095 Jiefang Avenue, Qiaokou District, Wuhan, 430030 China

**Keywords:** Staged, Health education pathway, Checklist, Esophageal cancer patients

## Abstract

**Objective:**

To evaluate the effectiveness of a staged health education pathway in the perioperative care of patients with esophageal cancer.

**Methods:**

A total of 110 patients who underwent radical surgery for esophageal cancer and were discharged from our department between January and November 2022 were enrolled in the study. Patients were divided into a control group and an experimental group (55 patients each) based on the order of admission. The control group received routine health education, in which the responsible nurses provided subjective and random health guidance based on patients’ daily treatment and condition. The experimental group received education based on a standardized staged health education pathway checklist. The two groups were compared in terms of discharge readiness scores and health education effectiveness, assessed using the Health Education Evaluation Criteria for Thoracic Surgery Patients.

**Results:**

The discharge readiness scores in the experimental group were significantly higher than those in the control group (*P* < 0.01). In addition, the experimental group outperformed the control group in overall health education scores and in the dimensions of health knowledge, health beliefs, and health behaviors, with statistically significant differences (*P* < 0.01).

**Conclusion:**

The application of a standardized staged health education pathway checklist in the perioperative period of esophageal cancer patients allows nurses to provide more systematic and timely health guidance. This approach helps patients and their families gain a more comprehensive understanding of the disease and better meets their health information needs.

## Introduction

Esophageal carcinoma (EC) is one of the most common malignant tumors encountered in clinical practice. According to the International Agency for Research on Cancer (IARC) under the World Health Organization, China bears the heaviest burden of EC worldwide, with an overall 5-year survival rate ranging from 15% to 40% [1]. At present, surgical intervention remains one of the most effective treatment options for EC [2]. In recent years, increasing emphasis has been placed on improving the quality and scope of specialized nursing services, with health education recognized as a vital element of holistic, responsibility-driven patient care [3]. This shift reflects a broader commitment within healthcare systems to strengthen patient-centered practices and promote more effective perioperative support.

Patients with EC typically undergo major surgical procedures associated with significant trauma and a high risk of postoperative complications, making perioperative health education especially critical. Previous domestic and international studies have explored the application of structured health education pathways in perioperative care and have reported benefits such as enhanced patient understanding, improved communication between healthcare providers and patients, and better adherence to treatment instructions [4–7]. While these studies do not directly measure improvements in clinical endpoints such as complication rates or surgical safety indicators, they provide supporting evidence for the potential of structured education to positively influence patient engagement and care processes, which are indirectly related to treatment quality and safety.

A Health Education Pathway (HEP) is a structured approach modeled on the concept of clinical pathways [8, 9], specifically designed to guide health education in nursing practice by outlining clear, time-phased, and disease-specific educational content. For patients with various medical conditions, the HEP delineates the necessary health education actions, their timing, sequence, and implementation strategies. By offering a concise written checklist, it enables patients to understand the expected educational process, stay informed of their schedule, and actively participate in their care. This structured approach not only enhances patients’ understanding of their illness but also promotes adherence to postoperative instructions, strengthens self-care abilities, and ultimately contributes to fewer complications, quicker recovery, and improved quality of life. Notably, our hospital—being a high-volume national esophageal cancer treatment center—receives patient referrals from multiple provinces. A regional screening and early detection initiative further supports the high rate of patient diagnosis and admission. These factors have enabled us to gather a substantial patient cohort within a relatively short period, providing a strong foundation for evaluating the effectiveness of the staged health education pathway model in clinical settings.

This study explores the application of a staged health education pathway checklist during the perioperative period in patients undergoing surgery for EC. The goal is to meet patients’ evolving health information needs through a targeted and timely education process, empowering them to take an active role in their recovery and long-term disease management.

## Methods

### Patients and procedure

**Fig. 1:**
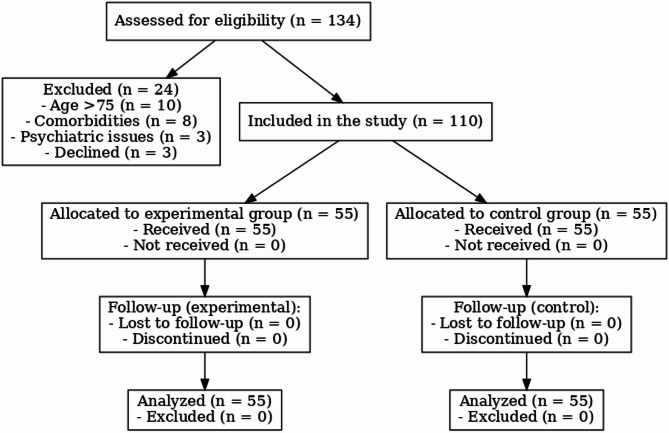
CONSORT-style flow diagram illustrating the process of patient enrollment, group allocation, follow-up, and data analysis

A total of 110 patients who underwent radical esophagectomy for EC and were discharged from our department between January 2021 and November 2021 were enrolled in this study. Based on the order of admission, patients were assigned to either a control group or an experimental group, with 55 patients in each. Inclusion criteria were: (i) age ≤ 75 years (as elderly patients over 75 often present with age-related physiological decline, multiple comorbidities, and reduced cognitive or physical function, which may interfere with compliance to structured health education and postoperative recovery pathways); (ii) normal verbal communication and comprehension abilities; (iii) histologically confirmed diagnosis of EC and scheduled for elective radical surgery; (iv) first-time surgical patients; (v) voluntary participation with signed informed consent. Exclusion criteria were: (i) presence of major comorbid diseases in other organs; (ii) diagnosed psychiatric disorders or impaired consciousness. The CONSORT-style flow diagram to visually depict the process of patient enrollment, group allocation, follow-up, and data analysis is in Fig. 1.

The sample size was calculated based on a two-tailed test with a significance level of α = 0.05, power = 80% (β = 0.20), and an estimated effect size (Cohen’s d) of 0.5, representing a medium effect. Using the standard formula: $\mathrm{n=2}\left(\mathrm Z1-\alpha/2+\mathrm Z1-\upbeta\right)2\cdot\mathrm\sigma2/\mathrm\delta2$, where σ is the standard deviation and δ is the mean difference to be detected, the required sample size per group was determined to be 51 participants. Accounting for a potential 5–10% dropout rate, we enrolled 55 participants in each group.

Clinical data included patients’ age, gender, education level, and admission date. All patients had pathologically confirmed EC, were undergoing their first elective radical esophagectomy, and had normal communication abilities. Upon admission, a structured assessment covered basic health status, hospital orientation, and infection control. Health education was delivered in stages throughout hospitalization, including preoperative preparation, surgical day instructions, postoperative recovery guidance, and discharge planning. Education focused on respiratory training, nutrition, complication prevention, functional recovery, medication adherence, and follow-up, with continued support provided through a WeChat group after discharge.

In this study, patients were allocated to the control or experimental group in an alternating sequence based on their order of admission (i.e., patients admitted in odd-numbered order were assigned to the control group, and even-numbered patients to the experimental group) to evaluate the effectiveness of a staged health education pathway during the perioperative period of EC surgery. The control group received routine health education in accordance with standard nursing practices for thoracic surgery. Upon admission, nurses conducted basic intake procedures, provided orientation to the ward environment and hospital policies, and introduced the medical care team. Prior to surgery, patients received examination appointment forms and were reminded daily to follow a respiratory training video produced by the department. Education before surgery also included guidance on fasting, personal preparation, and expectations for the surgical process. After surgery, patients and caregivers were educated on respiratory exercises, coughing and sputum clearance techniques, drainage tube care, and nutritional support. At discharge, they were given instructions on follow-up, billing procedures, and invited to join a departmental WeChat group for continued support.

The experimental group received the same routine education, supplemented by a structured staged health education pathway checklist. To develop this tool, a team of two associate chief nurses and five senior nurses, each with 10–25 years of thoracic surgery experience, conducted an extensive literature review using databases such as CNKI, Wanfang, Chinese Medical Journals Full-text Database, and PubMed. Keywords included terms like “EC,” “health education,” and “perioperative care.” The team synthesized both domestic and international evidence to design a tailored, stage-specific health education pathway for EC patients.

After the checklist was finalized, all responsible nurses received standardized training and were assessed to ensure consistency in its implementation. The checklist detailed the timing, content, and sequence of health education interventions at each stage of the patients’ hospital stay. Patients in the experimental group received education based on this structured pathway, as shown in Table 1.

**Table 1:** Staged health education pathway checklist for perioperative EC patients

Patient Name	Ward	Admission Date	Discharge Date	ID No.	Educator Signature
Hospitalization Day 1: Admission Health Education Assessment					
Health Education Content		Completion Status			Educator Signature
Personnel: Physician, Head Nurse, Responsible Nurse					
Environment: Various work areas					
System: Infection prevention protocols					
Evaluation: Basic assessment scale					
Education: Various admission instructions					
Hospitalization Days 2–7					
Health Education Content		Completion Status			Educator Signature
Introduction to preoperative exams					
Preoperative rehab training: Pulmonary function exercise video					
Preoperative fasting instructions					
Bowel preparation instructions and methods					
Explanation of surgical procedure and anesthesia					
Surgical instruments introduction					
Preoperative dietary guidelines					
Personal hygiene and preparation					
Preoperative medication instructions					
Surgical risk education					
Explanation of postoperative nursing procedures (ECG monitoring, oxygen therapy)					
Introduction to postoperative function recovery exercises					
Medication administration route and sources					
Hospitalization Days 9–14: Postoperative Days 1–7					
Health Education Content		Completion Status			Educator Signature
Comfort Education:					
- Pain management guidance					
- Psychological care					
- Non-drug pain relief methods					
- Post-op body positioning guidance					
- Wound care education					
- Complication prevention					
Pulmonary Function Training:					
- Pulmonary function recovery exercises					
- Breathing training					
- Effective cough and sputum expectoration techniques					
- Nebulized inhalation therapy					
Nutritional Rehabilitation Education:					
- Intestinal nutrition precautions					
- Parenteral nutrition precautions					
- Swallowing training					
- Drinking water training					
Hospitalization Days 15–22: Postoperative Days 8–14					
Health Education Content		Completion Status			Educator Signature
Oral intake progression instructions					
Discharge education (with nutrition discharge instructions)					
Healthy lifestyle guidance					
Post-discharge dietary guidance					
Discharge Day					
Health Education Content		Completion Status			Educator Signature
Post-discharge dietary guidance					
Post-discharge complication prevention					
Post-discharge rehabilitation (including limb function training, respiratory recovery, swallowing training)					
Medication instructions after discharge					
Important points for follow-up visits					
Join the WeChat group for continued care after thoracic surgery					
Hospital discharge process					

### Evaluation indicators

The Readiness for Hospital Discharge Scale (RHDS), developed by Weiss et al. [10] based on Meleis’s Transition Theory, is widely used to assess patients’ readiness for discharge across various clinical settings. In this study, we used the Chinese version adapted by Taiwanese scholar Yu Q et al. [11]., which was culturally localized to align with both Eastern and Western perspectives. This is a self-report scale comprising three dimensions: personal status (3 items), coping ability (5 items), and anticipated support (4 items), for a total of 12 items. The first item, “Are you ready to be discharged?” is a yes/no question and is not included in the total score. The remaining 11 items are rated on a scale from 0 (not at all prepared) to 10 (fully prepared), yielding a total score range of 0–110. Higher scores indicate a greater level of discharge readiness. Each dimension’s average score is calculated by dividing the total score of that dimension by the number of items it contains. The scale has demonstrated good reliability and validity, with a Cronbach’s alpha of 0.89 and a content validity index (CVI) of 0.88; individual item CVIs range from 0.80 to 1.00.

### Health education evaluation criteria for thoracic surgery patients

This scale was used to compare the health education outcomes between the two patient groups. The scale is based on the “Knowledge–Belief–Practice” (KBP) theoretical model of health education [12]. It incorporates the structure of the American Marion Nursing Evaluation Classification System [3] and integrates years of clinical and research experience in thoracic surgery. The tool evaluates health education effectiveness across three dimensions: health knowledge (25 items), health beliefs (6 items), and health behaviors (12 items), totaling 43 items. Each item is scored on a 4-point Likert scale: 1 (not proactive), 2 (somewhat passive), 3 (fairly proactive), and 4 (very proactive). The total score ranges from 43 to 172, with higher scores indicating better health education outcomes. Evaluation is conducted through patient questioning, observation, and assessment of their recall and demonstration of the educational content.

### Statistical analysis

All data were analyzed using SPSS version 26.0. Independent-samples t-tests were applied to compare the RHDS scores and the health education evaluation scores between the two groups. We conducted Shapiro–Wilk tests for normality. If data were non-normally distributed, we used Mann–Whitney U tests accordingly. A *p*-value of less than 0.05 was considered statistically significant.

## Results

### Comparison of discharge readiness scores between the two groups

Table 2 presents a comparison of discharge readiness scores between the control and experimental groups, evaluated using the RHDS. The results demonstrate that patients in the experimental group, who received structured, staged health education guided by a checklist, exhibited significantly higher levels of discharge readiness across all dimensions. In the dimension of personal status, the experimental group scored 23.709 ± 2.872, markedly higher than the control group’s 13.000 ± 3.043. Similarly, for coping ability, the experimental group scored 37.782 ± 2.183, compared to 29.182 ± 3.678 in the control group. In terms of anticipated support, scores were 36.691 ± 1.275 and 25.927 ± 2.062 for the experimental and control groups, respectively. The total readiness score was also significantly higher in the experimental group (90.655 ± 3.888) than in the control group (58.618 ± 4.994). All differences were statistically significant (*P* < 0.001). These findings indicate that the use of a structured, phased health education pathway significantly improves patients’ preparedness for discharge, enhancing their physical and emotional readiness, coping capacity, and perception of available support after leaving the hospital.

**Table 2:** Comparison of discharge readiness scores between the two groups

Dimension	Control Group (*n* = 55)	Experimental Group (*n* = 55)	t-value	*P*-value
Dimension 1: Personal Status	13.000 ± 3.043	23.709 ± 2.872	−18.982	< 0.001
Dimension 2: Coping Ability	29.182 ± 3.678	37.782 ± 2.183	−14.914	< 0.001
Dimension 3: Anticipated Support	25.927 ± 2.062	36.691 ± 1.275	−32.923	< 0.001
Total Readiness Score	58.618 ± 4.994	90.655 ± 3.888	−37.536	< 0.001

### Improved health education outcomes through staged educational interventions

Table 3 presents a comparison of health education evaluation scores between the control and experimental groups. The results demonstrate that patients in the experimental group, who received structured, staged health education based on a checklist, achieved significantly higher scores across all dimensions of the evaluation. The total score for health education effectiveness in the experimental group was 143.418 ± 6.005, notably higher than the control group’s score of 102.072 ± 12.893. In the dimension of health knowledge, the experimental group scored 91.836 ± 4.467, compared to 64.782 ± 2.846 in the control group. For health beliefs, the experimental group reached a score of 21.509 ± 4.417, whereas the control group scored 12.600 ± 3.837. In terms of health behaviors, the experimental group scored 34.255 ± 2.496, significantly exceeding the control group’s score of 25.182 ± 1.701. All differences between the two groups were statistically significant (*P* < 0.001). These findings suggest that the implementation of a staged health education pathway significantly improved patients’ understanding of health-related knowledge, strengthened their health beliefs, and promoted more proactive health behaviors.

**Table 3:** Comparison of health education evaluation scores between the two groups

Item	Control Group	Experimental Group	t-value	*P*-value
Total Health Education Score	102.072 ± 12.893	143.418 ± 6.005	−21.557	< 0.001
Dimension 1: Health Knowledge	64.782 ± 2.846	91.836 ± 4.467	−37.881	< 0.001
Dimension 2: Health Beliefs	12.600 ± 3.837	21.509 ± 4.417	−11.291	< 0.001
Dimension 3: Health Behaviors	25.182 ± 1.701	34.255 ± 2.496	−22.277	< 0.001

## Discussion

Readiness for hospital discharge reflects both a healthcare provider’s evaluation of a patient’s ability to continue recovery at home and the patient’s own perception of being prepared to leave the hospital [13, 14]. While discharge is often determined by physicians, under the Enhanced Recovery After Surgery (ERAS) concept, greater emphasis should be placed on the patient’s experience and readiness during hospitalization. For patients undergoing esophagectomy, discharge marks the transition back to home and society, where ongoing care and treatment are typically required. Therefore, discharge readiness is a critical indicator of a patient’s basic recovery status and their ability to safely leave the hospital [15]. It is also vital for reducing complications and readmission rates following surgery.

In this study, the experimental group began receiving staged health education through a structured checklist from the time of admission. This systematic education covered pulmonary rehabilitation, swallowing exercises, effective coughing techniques, and enteral nutrition, complemented by department-specific instructional videos. Nurses provided real-time guidance during routine care, meeting patients’ and families’ informational needs and encouraging active participation. This engagement significantly improved patients’ health behaviors and enhanced their readiness for discharge. Existing research supports this finding, showing that higher discharge readiness is associated with improved post-discharge self-care, fewer unplanned readmissions, and lower rates of complications and healthcare costs [16]. Our results confirmed this trend, with the experimental group demonstrating significantly higher discharge readiness scores than the control group [17]. The staged checklist served as a structured timeline that standardized health education delivery, ensuring patients were well-prepared for discharge and better equipped for home care.

Health education is a crucial component of perioperative care, especially for EC patients undergoing ERAS protocols [18]. It promotes patient engagement in recovery and contributes to improved surgical outcomes. The formation of health behaviors is closely tied to patients’ beliefs and knowledge [19]. By implementing the staged health education pathway checklist, nurses were able to deliver timely and comprehensive education during routine care, ensuring no important information was missed.

From the dimension of health knowledge, patients in the experimental group scored significantly higher than those in the control group. The standardized educational materials in this study replaced traditional nurse-centered, subjective methods with structured content and timely patient assessments. This approach enhances patients’ understanding of perioperative care [18]. The checklist addressed common gaps and misconceptions about disease knowledge, including key postoperative concerns such as swallowing function, nutrition, and eating habits.

In terms of health beliefs, the experimental group again outperformed the control group. Building on improved knowledge, the checklist allowed nurses to deliver targeted daily education based on each patient’s progress, such as meal volume and eating speed—factors that are especially important to EC patients and strongly linked to discharge readiness. Clear, consistent messaging from nurses encouraged patient compliance and fostered a proactive attitude toward health, shifting patients from passive recipients to active participants in their care [20].

Regarding health behaviors, the experimental group also scored significantly higher. The ultimate goal of health education is to promote actual behavioral change [21]. In the experimental group, the highest scoring behaviors included strict smoking cessation, seeking postoperative exercise guidance, and early mobilization. While both groups valued smoking cessation, patients in the control group often performed exercises incorrectly despite watching educational videos, which led to ineffective recovery time [22]. In contrast, patients in the experimental group actively sought guidance on rehabilitation techniques and mobility. Nurses responded with timely instructions, including on pain management, which reduced discomfort and encouraged movement. This approach aligns with findings that pathway-based education improves recovery outcomes [23].

Given the complexity and continuity of postoperative education for EC patients, variation in timing and method of instruction often exists. The staged health education checklist clearly outlines content and allows nurses to self-audit their delivery. This ensures education is guided, thorough, and standardized. Moreover, the checklist improves nurses’ service mindset, enhances their professional knowledge, and reduces the risk of inadequate education due to lack of experience. Although our findings demonstrate that the staged health education pathway significantly improves patients’ perceived readiness for discharge and health knowledge, these results primarily reflect patient-reported outcomes rather than objective clinical endpoints.

This study has several limitations that should be acknowledged. First, a potential source of bias exists in the experimental group, as the same nursing staff who delivered the staged health education intervention were also responsible for evaluating patient outcomes. The absence of blinding in this process represents a methodological weakness and may compromise the objectivity of the results. Second, the patient allocation method was based on the order of admission rather than true randomization, which may introduce selection bias despite efforts to match group characteristics. Third, the exclusion of patients over 75 years of age and those with significant comorbidities, while necessary to reduce confounding factors and ensure patient compliance, may limit the generalizability of the findings to broader esophageal cancer populations. Furthermore, our study focused on perceived outcomes rather than objective clinical endpoints (e.g., complication rates, length of stay, readmission), and therefore cannot definitively conclude that structured education leads to improved health outcomes. Lastly, the study was conducted in a single high-volume cancer center, and the results may not be fully applicable to institutions with different patient volumes, healthcare systems, or educational resources. Future multi-center studies with blinded assessments and more diverse patient populations are needed to further validate these findings.

## Conclusion

EC surgery is a major life event for patients and their families, often causing psychological distress. Comprehensive perioperative education is essential. The staged health education pathway checklist standardizes the content and improves the quality of health education, fostering active participation from patients and their families. This not only strengthens the nurse-patient relationship but also promotes a sense of being valued. Continued refinement of the checklist, with a focus on patient-centered care and lived experience, will further support recovery in EC patients.

## Data Availability

The datasets generated and analyzed during the current study are available from the corresponding author on reasonable request.

## Abbreviations

EC: Esophageal carcinoma
IARC: International Agency for Research on Cancer
HEP: Health Education Pathway
RHDS: The Readiness for Hospital Discharge Scale
